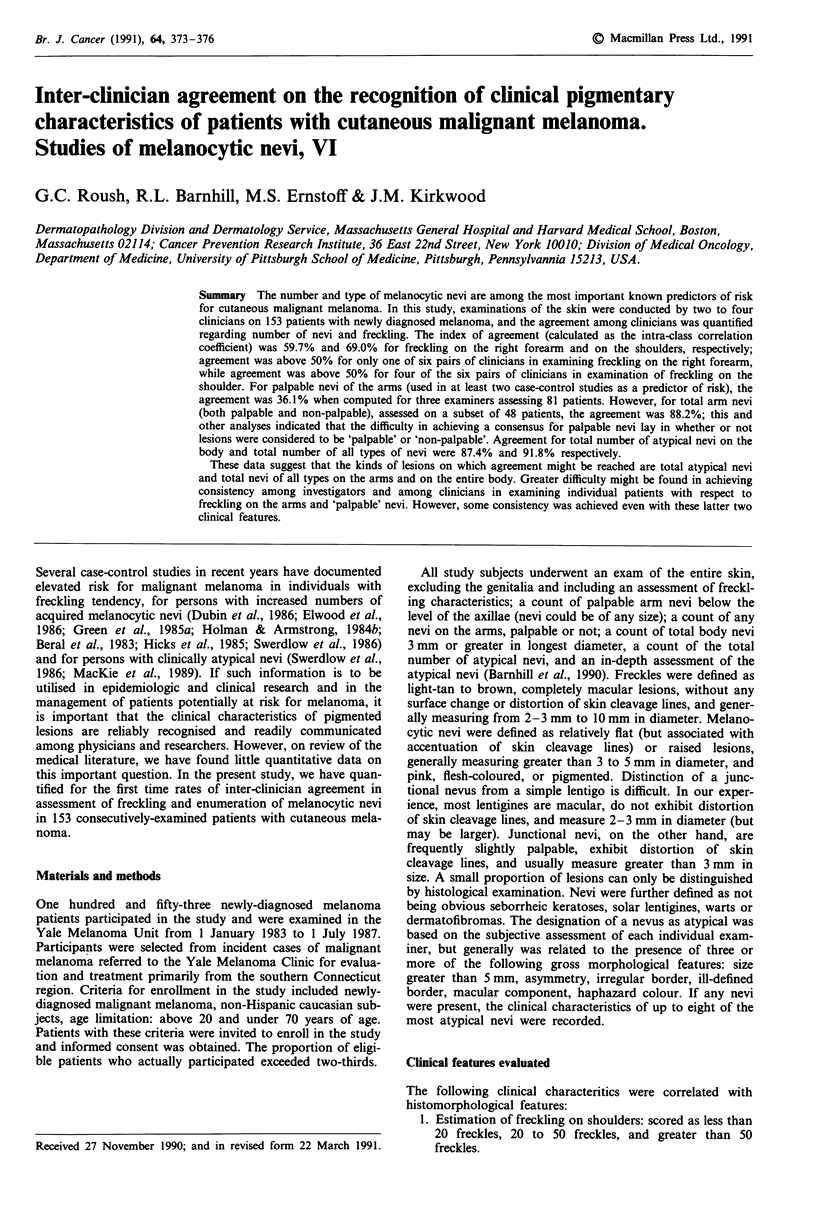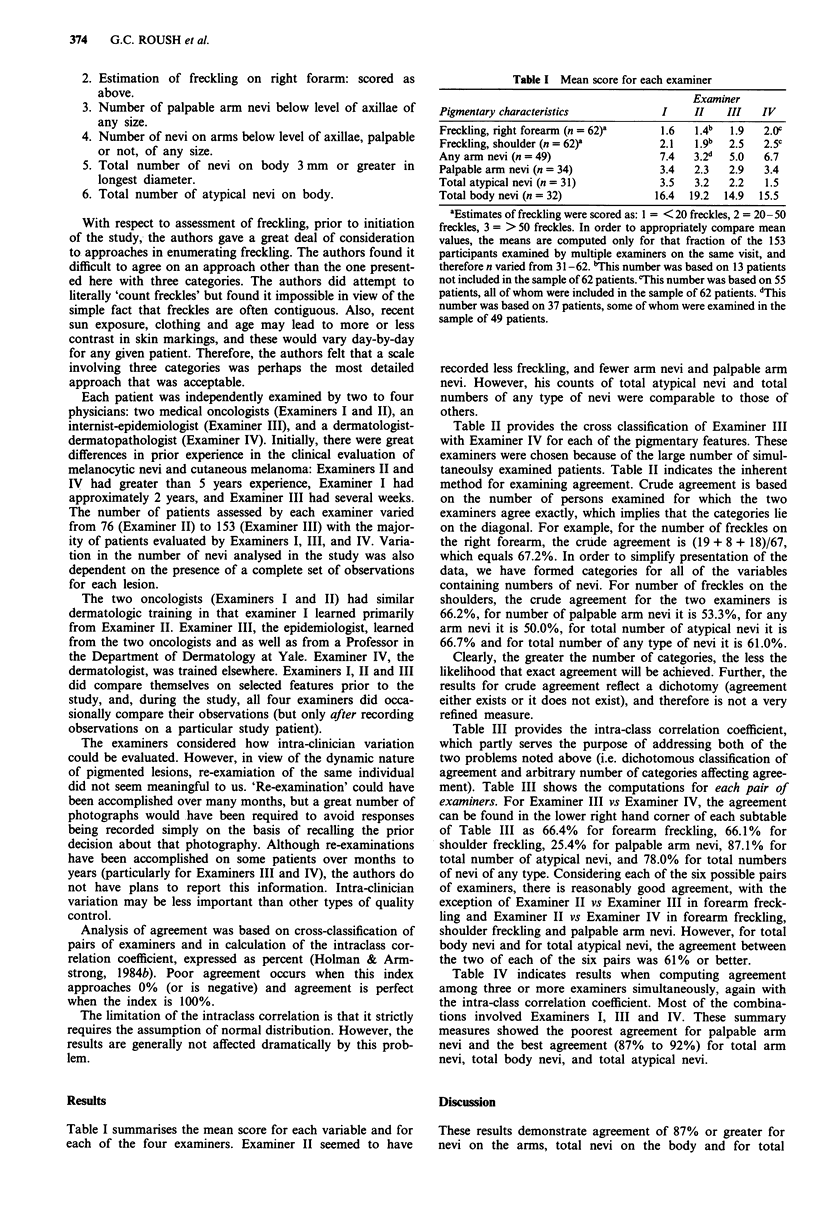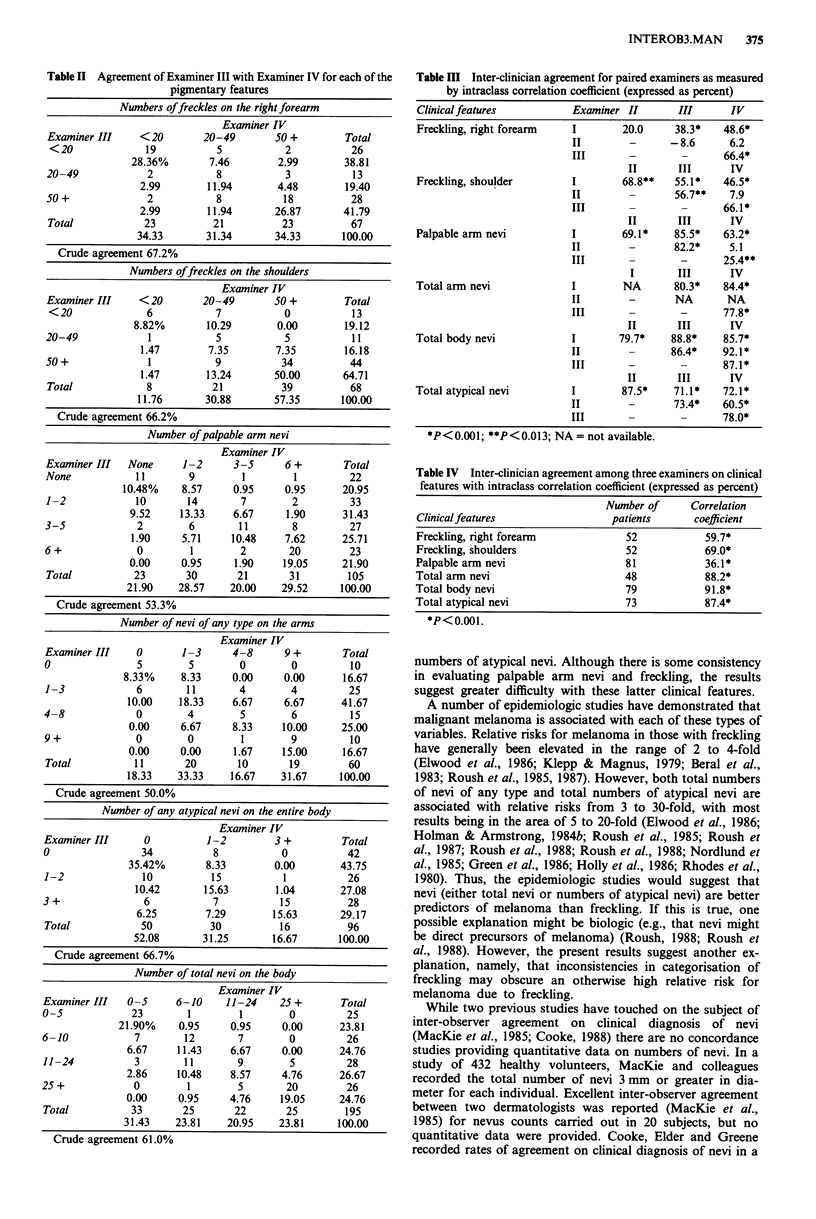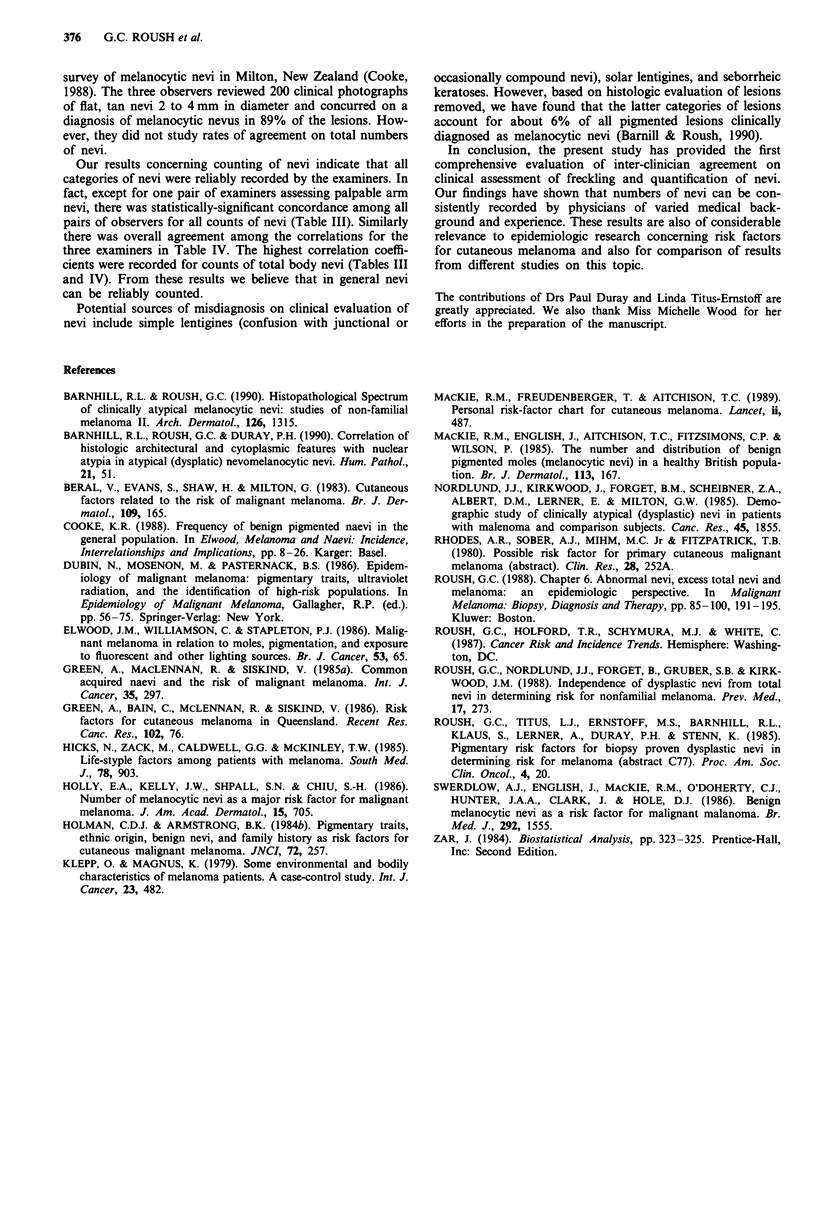# Inter-clinician agreement on the recognition of clinical pigmentary characteristics of patients with cutaneous malignant melanoma. Studies of melanocytic nevi, VI.

**DOI:** 10.1038/bjc.1991.310

**Published:** 1991-08

**Authors:** G. C. Roush, R. L. Barnhill, M. S. Ernstoff, J. M. Kirkwood

**Affiliations:** Dermatopathology Division and Dermatology Service, Massachusetts General Hospital and Harvard Medical School, Boston 02114.

## Abstract

The number and type of melanocytic nevi are among the most important known predictors of risk for cutaneous malignant melanoma. In this study, examinations of the skin were conducted by two to four clinicians on 153 patients with newly diagnosed melanoma, and the agreement among clinicians was quantified regarding number of nevi and freckling. The index of agreement (calculated as the intra-class correlation coefficient) was 59.7% and 69.0% for freckling on the right forearm and on the shoulders, respectively; agreement was above 50% for only one of six pairs of clinicians in examining freckling on the right forearm, while agreement was above 50% for four of the six pairs of clinicians in examination of freckling on the shoulder. For palpable nevi of the arms (used in at least two case-control studies as a predictor of risk), the agreement was 36.1% when computed for three examiners assessing 81 patients. However, for total arm nevi (both palpable and non-palpable), assessed on a subset of 48 patients, the agreement was 88.2%; this and other analyses indicated that the difficulty in achieving a consensus for palpable nevi lay in whether or not lesions were considered to be 'palpable' or 'non-palpable'. Agreement for total number of atypical nevi on the body and total number of all types of nevi were 87.4% and 91.8% respectively. These data suggest that the kinds of lesions on which agreement might be reached are total atypical nevi and total nevi of all types on the arms and on the entire body. Greater difficulty might be found in achieving consistency among investigators and among clinicians in examining individual patients with respect to freckling on the arms and 'palpable' nevi. However, some consistency was achieved even with these latter two clinical features.


*Br. J. Cancer* (1991), **64**, 373–376 © Macmillan Press Ltd., 1991

### Inter-clinician agreement on the recognition of clinical pigmentary characteristics of patients with cutaneous malignant melanoma. Studies of melanocytic nevi, VI

G.C. Roush, R.L. Barnhill, M.S. Ernstoff & J.M. Kirkwood

*Dermatopathology Division and Dermatology Service, Massachusetts General Hospital and Harvard Medical School, Boston, Massachusetts 02114; Cancer Prevention Research Institute, 36 East 22nd Street, New York 10010; Division of Medical Oncology, Department of Medicine, University of Pittsburgh School of Medicine, Pittsburgh, Pennsylvannia 15213, USA.*

**Summary** The number and type of melanocytic nevi are among the most important known predictors of risk for cutaneous malignant melanoma. In this study, examinations of the skin were conducted by two to four clinicians on 153 patients with newly diagnosed melanoma, and the agreement among clinicians was quantified regarding number of nevi and freckling. The index of agreement (calculated as the intra-class correlation coefficient) was 59.7% and 69.0% for freckling on the right forearm and on the shoulders, respectively; agreement was above 50% for only one of six pairs of clinicians in examining freckling on the right forearm, while agreement was above 50% for four of the six pairs of clinicians in examination of freckling on the shoulder. For palpable nevi of the arms (used in at least two case-control studies as a predictor of risk), the agreement was 36.1% when computed for three examiners assessing 81 patients. However, for total arm nevi (both palpable and non-palpable), assessed on a subset of 48 patients, the agreement was 88.2%; this and other analyses indicated that the difficulty in achieving a consensus for palpable nevi lay in whether or not lesions were considered to be 'palpable' or 'non-palpable'. Agreement for total number of atypical nevi on the body and total number of all types of nevi were 87.4% and 91.8% respectively.

These data suggest that the kinds of lesions on which agreement might be reached are total atypical nevi and total nevi of all types on the arms and on the entire body. Greater difficulty might be found in achieving consistency among investigators and among clinicians in examining individual patients with respect to freckling on the arms and 'palpable' nevi. However, some consistency was achieved even with these latter two clinical features.

Several case-control studies in recent years have documented elevated risk for malignant melanoma in individuals with freckling tendency, for persons with increased numbers of acquired melanocytic nevi (Dubin *et al.*, 1986; Elwood *et al.*, 1986; Green *et al.*, 1985*a*; Holman & Armstrong, 1984*b*; Beral *et al.*, 1983; Hicks *et al.*, 1985; Swerdlow *et al.*, 1986) and for persons with clinically atypical nevi (Swerdlow *et al.*, 1986; MacKie *et al.*, 1989). If such information is to be utilised in epidemiologic and clinical research and in the management of patients potentially at risk for melanoma, it is important that the clinical characteristics of pigmented lesions are reliably recognised and readily communicated among physicians and researchers. However, on review of the medical literature, we have found little quantitative data on this important question. In the present study, we have quantified for the first time rates of inter-clinician agreement in assessment of freckling and enumeration of melanocytic nevi in 153 consecutively-examined patients with cutaneous melanoma.

### Materials and methods

One hundred and fifty-three newly-diagnosed melanoma patients participated in the study and were examined in the Yale Melanoma Unit from 1 January 1983 to 1 July 1987. Participants were selected from incident cases of malignant melanoma referred to the Yale Melanoma Clinic for evaluation and treatment primarily from the southern Connecticut region. Criteria for enrollment in the study included newly-diagnosed malignant melanoma, non-Hispanic caucasian subjects, age limitation: above 20 and under 70 years of age. Patients with these criteria were invited to enroll in the study and informed consent was obtained. The proportion of eligible patients who actually participated exceeded two-thirds.

Received 27 November 1990; and in revised form 22 March 1991.

All study subjects underwent an exam of the entire skin, excluding the genitalia and including an assessment of freckling characteristics; a count of palpable arm nevi below the level of the axillae (nevi could be of any size); a count of any nevi on the arms, palpable or not; a count of total body nevi 3 mm or greater in longest diameter, a count of the total number of atypical nevi, and an in-depth assessment of the atypical nevi (Barnhill *et al.*, 1990). Freckles were defined as light-tan to brown, completely macular lesions, without any surface change or distortion of skin cleavage lines, and generally measuring from 2–3 mm to 10 mm in diameter. Melanocytic nevi were defined as relatively flat (but associated with accentuation of skin cleavage lines) or raised lesions, generally measuring greater than 3 to 5 mm in diameter, and pink, flesh-coloured, or pigmented. Distinction of a junctional nevus from a simple lentigo is difficult. In our experience, most lentigines are macular, do not exhibit distortion of skin cleavage lines, and measure 2–3 mm in diameter (but may be larger). Junctional nevi, on the other hand, are frequently slightly palpable, exhibit distortion of skin cleavage lines, and usually measure greater than 3 mm in size. A small proportion of lesions can only be distinguished by histological examination. Nevi were further defined as not being obvious seborrheic keratoses, solar lentigines, warts or dermatofibromas. The designation of a nevus as atypical was based on the subjective assessment of each individual examiner, but generally was related to the presence of three or more of the following gross morphological features: size greater than 5 mm, asymmetry, irregular border, ill-defined border, macular component, haphazard colour. If any nevi were present, the clinical characteristics of up to eight of the most atypical nevi were recorded.

### Clinical features evaluated

The following clinical characteritics were correlated with histomorphological features:

1. Estimation of freckling on shoulders: scored as less than 20 freckles, 20 to 50 freckles, and greater than 50 freckles.

2. Estimation of freckling on right forarm: scored as above.
3. Number of palpable arm nevi below level of axillae of any size.
4. Number of nevi on arms below level of axillae, palpable or not, of any size.
5. Total number of nevi on body 3 mm or greater in longest diameter.
6. Total number of atypical nevi on body.

With respect to assessment of freckling, prior to initiation of the study, the authors gave a great deal of consideration to approaches in enumerating freckling. The authors found it difficult to agree on an approach other than the one presented here with three categories. The authors did attempt to literally 'count freckles' but found it impossible in view of the simple fact that freckles are often contiguous. Also, recent sun exposure, clothing and age may lead to more or less contrast in skin markings, and these would vary day-by-day for any given patient. Therefore, the authors felt that a scale involving three categories was perhaps the most detailed approach that was acceptable.

Each patient was independently examined by two to four physicians: two medical oncologists (Examiners I and II), an internist-epidemiologist (Examiner III), and a dermatologist-dermatopathologist (Examiner IV). Initially, there were great differences in prior experience in the clinical evaluation of melanocytic nevi and cutaneous melanoma: Examiners II and IV had greater than 5 years experience, Examiner I had approximately 2 years, and Examiner III had several weeks. The number of patients assessed by each examiner varied from 76 (Examiner II) to 153 (Examiner III) with the majority of patients evaluated by Examiners I, III, and IV. Variation in the number of nevi analysed in the study was also dependent on the presence of a complete set of observations for each lesion.

The two oncologists (Examiners I and II) had similar dermatologic training in that examiner I learned primarily from Examiner II. Examiner III, the epidemiologist, learned from the two oncologists and as well as from a Professor in the Department of Dermatology at Yale. Examiner IV, the dermatologist, was trained elsewhere. Examiners I, II and III did compare themselves on selected features prior to the study, and, during the study, all four examiners did occasionally compare their observations (but only *after* recording observations on a particular study patient).

The examiners considered how intra-clinician variation could be evaluated. However, in view of the dynamic nature of pigmented lesions, re-examiation of the same individual did not seem meaningful to us. 'Re-examination' could have been accomplished over many months, but a great number of photographs would have been required to avoid responses being recorded simply on the basis of recalling the prior decision about that photography. Although re-examinations have been accomplished on some patients over months to years (particularly for Examiners III and IV), the authors do not have plans to report this information. Intra-clinician variation may be less important than other types of quality control.

Analysis of agreement was based on cross-classification of pairs of examiners and in calculation of the intraclass correlation coefficient, expressed as percent (Holman & Armstrong, 1984*b*). Poor agreement occurs when this index approaches 0% (or is negative) and agreement is perfect when the index is 100%.

The limitation of the intraclass correlation is that it strictly requires the assumption of normal distribution. However, the results are generally not affected dramatically by this problem.

### Results

Table I summarises the mean score for each variable and for each of the four examiners. Examiner II seemed to have recorded less freckling, and fewer arm nevi and palpable arm nevi. However, his counts of total atypical nevi and total numbers of any type of nevi were comparable to those of others.

**Table I** Mean score for each examiner

| *Pigmentary characteristics* | Examiner *I* | *II* | *III* | *IV* |
|---|---|---|---|---|
| Freckling, right forearm ($n = 62$)[a] | 1.6 | 1.4[b] | 1.9 | 2.0[c] |
| Freckling, shoulder ($n = 62$)[a] | 2.1 | 1.9[b] | 2.5 | 2.5[c] |
| Any arm nevi ($n = 49$) | 7.4 | 3.2[d] | 5.0 | 6.7 |
| Palpable arm nevi ($n = 34$) | 3.4 | 2.3 | 2.9 | 3.4 |
| Total atypical nevi ($n = 31$) | 3.5 | 3.2 | 2.2 | 1.5 |
| Total body nevi ($n = 32$) | 16.4 | 19.2 | 14.9 | 15.5 |

[a]Estimates of freckling were scored as: 1 = <20 freckles, 2 = 20–50 freckles, 3 = >50 freckles. In order to appropriately compare mean values, the means are computed only for that fraction of the 153 participants examined by multiple examiners on the same visit, and therefore $n$ varied from 31–62. [b]This number was based on 13 patients not included in the sample of 62 patients. [c]This number was based on 55 patients, all of whom were included in the sample of 62 patients. [d]This number was based on 37 patients, some of whom were examined in the sample of 49 patients.

Table II provides the cross classification of Examiner III with Examiner IV for each of the pigmentary features. These examiners were chosen because of the large number of simultaneoulsy examined patients. Table II indicates the inherent method for examining agreement. Crude agreement is based on the number of persons examined for which the two examiners agree exactly, which implies that the categories lie on the diagonal. For example, for the number of freckles on the right forearm, the crude agreement is $(19 + 8 + 18)/67$, which equals 67.2%. In order to simplify presentation of the data, we have formed categories for all of the variables containing numbers of nevi. For number of freckles on the shoulders, the crude agreement for the two examiners is 66.2%, for number of palpable arm nevi it is 53.3%, for any arm nevi it is 50.0%, for total number of atypical nevi it is 66.7% and for total number of any type of nevi it is 61.0%.

Clearly, the greater the number of categories, the less the likelihood that exact agreement will be achieved. Further, the results for crude agreement reflect a dichotomy (agreement either exists or it does not exist), and therefore is not a very refined measure.

Table III provides the intra-class correlation coefficient, which partly serves the purpose of addressing both of the two problems noted above (i.e. dichotomous classification of agreement and arbitrary number of categories affecting agreement). Table III shows the computations for *each pair of examiners*. For Examiner III *vs* Examiner IV, the agreement can be found in the lower right hand corner of each subtable of Table III as 66.4% for forearm freckling, 66.1% for shoulder freckling, 25.4% for palpable arm nevi, 87.1% for total number of atypical nevi, and 78.0% for total numbers of nevi of any type. Considering each of the six possible pairs of examiners, there is reasonably good agreement, with the exception of Examiner II *vs* Examiner III in forearm freckling and Examiner II *vs* Examiner IV in forearm freckling, shoulder freckling and palpable arm nevi. However, for total body nevi and for total atypical nevi, the agreement between the two of each of the six pairs was 61% or better.

Table IV indicates results when computing agreement among three or more examiners simultaneously, again with the intra-class correlation coefficient. Most of the combinations involved Examiners I, III and IV. These summary measures showed the poorest agreement for palpable arm nevi and the best agreement (87% to 92%) for total arm nevi, total body nevi, and total atypical nevi.

### Discussion

These results demonstrate agreement of 87% or greater for nevi on the arms, total nevi on the body and for total

**Table II** Agreement of Examiner III with Examiner IV for each of the pigmentary features

*Numbers of freckles on the right forearm*

| | Examiner IV | | | |
|---|---|---|---|---|
| *Examiner III* | *<20* | *20–49* | *50+* | *Total* |
| *<20* | 19 | 5 | 2 | 26 |
| | 28.36% | 7.46 | 2.99 | 38.81 |
| *20–49* | 2 | 8 | 3 | 13 |
| | 2.99 | 11.94 | 4.48 | 19.40 |
| *50+* | 2 | 8 | 18 | 28 |
| | 2.99 | 11.94 | 26.87 | 41.79 |
| *Total* | 23 | 21 | 23 | 67 |
| | 34.33 | 31.34 | 34.33 | 100.00 |

Crude agreement 67.2%

*Numbers of freckles on the shoulders*

| | Examiner IV | | | |
|---|---|---|---|---|
| *Examiner III* | *<20* | *20–49* | *50+* | *Total* |
| *<20* | 6 | 7 | 0 | 13 |
| | 8.82% | 10.29 | 0.00 | 19.12 |
| *20–49* | 1 | 5 | 5 | 11 |
| | 1.47 | 7.35 | 7.35 | 16.18 |
| *50+* | 1 | 9 | 34 | 44 |
| | 1.47 | 13.24 | 50.00 | 64.71 |
| *Total* | 8 | 21 | 39 | 68 |
| | 11.76 | 30.88 | 57.35 | 100.00 |

Crude agreement 66.2%

*Number of palpable arm nevi*

| | Examiner IV | | | | |
|---|---|---|---|---|---|
| *Examiner III* | *None* | *1–2* | *3–5* | *6+* | *Total* |
| *None* | 11 | 9 | 1 | 1 | 22 |
| | 10.48% | 8.57 | 0.95 | 0.95 | 20.95 |
| *1–2* | 10 | 14 | 7 | 2 | 33 |
| | 9.52 | 13.33 | 6.67 | 1.90 | 31.43 |
| *3–5* | 2 | 6 | 11 | 8 | 27 |
| | 1.90 | 5.71 | 10.48 | 7.62 | 25.71 |
| *6+* | 0 | 1 | 2 | 20 | 23 |
| | 0.00 | 0.95 | 1.90 | 19.05 | 21.90 |
| *Total* | 23 | 30 | 21 | 31 | 105 |
| | 21.90 | 28.57 | 20.00 | 29.52 | 100.00 |

Crude agreement 53.3%

*Number of nevi of any type on the arms*

| | Examiner IV | | | | |
|---|---|---|---|---|---|
| *Examiner III* | *0* | *1–3* | *4–8* | *9+* | *Total* |
| *0* | 5 | 5 | 0 | 0 | 10 |
| | 8.33% | 8.33 | 0.00 | 0.00 | 16.67 |
| *1–3* | 6 | 11 | 4 | 4 | 25 |
| | 10.00 | 18.33 | 6.67 | 6.67 | 41.67 |
| *4–8* | 0 | 4 | 5 | 6 | 15 |
| | 0.00 | 6.67 | 8.33 | 10.00 | 25.00 |
| *9+* | 0 | 0 | 1 | 9 | 10 |
| | 0.00 | 0.00 | 1.67 | 15.00 | 16.67 |
| *Total* | 11 | 20 | 10 | 19 | 60 |
| | 18.33 | 33.33 | 16.67 | 31.67 | 100.00 |

Crude agreement 50.0%

*Number of any atypical nevi on the entire body*

| | Examiner IV | | | |
|---|---|---|---|---|
| *Examiner III* | *0* | *1–2* | *3+* | *Total* |
| *0* | 34 | 8 | 0 | 42 |
| | 35.42% | 8.33 | 0.00 | 43.75 |
| *1–2* | 10 | 15 | 1 | 26 |
| | 10.42 | 15.63 | 1.04 | 27.08 |
| *3+* | 6 | 7 | 15 | 28 |
| | 6.25 | 7.29 | 15.63 | 29.17 |
| *Total* | 50 | 30 | 16 | 96 |
| | 52.08 | 31.25 | 16.67 | 100.00 |

Crude agreement 66.7%

*Number of total nevi on the body*

| | Examiner IV | | | | |
|---|---|---|---|---|---|
| *Examiner III* | *0–5* | *6–10* | *11–24* | *25+* | *Total* |
| *0–5* | 23 | 1 | 1 | 0 | 25 |
| | 21.90% | 0.95 | 0.95 | 0.00 | 23.81 |
| *6–10* | 7 | 12 | 7 | 0 | 26 |
| | 6.67 | 11.43 | 6.67 | 0.00 | 24.76 |
| *11–24* | 3 | 11 | 9 | 5 | 28 |
| | 2.86 | 10.48 | 8.57 | 4.76 | 26.67 |
| *25+* | 0 | 1 | 5 | 20 | 26 |
| | 0.00 | 0.95 | 4.76 | 19.05 | 24.76 |
| *Total* | 33 | 25 | 22 | 25 | 195 |
| | 31.43 | 23.81 | 20.95 | 23.81 | 100.00 |

Crude agreement 61.0%

**Table III** Inter-clinician agreement for paired examiners as measured by intraclass correlation coefficient (expressed as percent)

| *Clinical features* | *Examiner* | *II* | *III* | *IV* |
|---|---|---|---|---|
| Freckling, right forearm | I | 20.0 | 38.3* | 48.6* |
| | II | – | –8.6 | 6.2 |
| | III | – | – | 66.4* |
| | | II | III | IV |
| Freckling, shoulder | I | 68.8** | 55.1* | 46.5* |
| | II | – | 56.7** | 7.9 |
| | III | – | – | 66.1* |
| | | II | III | IV |
| Palpable arm nevi | I | 69.1* | 85.5* | 63.2* |
| | II | – | 82.2* | 5.1 |
| | III | – | – | 25.4** |
| | | I | III | IV |
| Total arm nevi | I | NA | 80.3* | 84.4* |
| | II | – | NA | NA |
| | III | – | – | 77.8* |
| | | II | III | IV |
| Total body nevi | I | 79.7* | 88.8* | 85.7* |
| | II | – | 86.4* | 92.1* |
| | III | – | – | 87.1* |
| | | II | III | IV |
| Total atypical nevi | I | 87.5* | 71.1* | 72.1* |
| | II | – | 73.4* | 60.5* |
| | III | – | – | 78.0* |

$^{*}P<0.001$; $^{**}P<0.013$; NA = not available.

**Table IV** Inter-clinician agreement among three examiners on clinical features with intraclass correlation coefficient (expressed as percent)

| *Clinical features* | *Number of patients* | *Correlation coefficient* |
|---|---|---|
| Freckling, right forearm | 52 | 59.7* |
| Freckling, shoulders | 52 | 69.0* |
| Palpable arm nevi | 81 | 36.1* |
| Total arm nevi | 48 | 88.2* |
| Total body nevi | 79 | 91.8* |
| Total atypical nevi | 73 | 87.4* |

$^{*}P<0.001$.

numbers of atypical nevi. Although there is some consistency in evaluating palpable arm nevi and freckling, the results suggest greater difficulty with these latter clinical features.

A number of epidemiologic studies have demonstrated that malignant melanoma is associated with each of these types of variables. Relative risks for melanoma in those with freckling have generally been elevated in the range of 2 to 4-fold (Elwood *et al.*, 1986; Klepp & Magnus, 1979; Beral *et al.*, 1983; Roush *et al.*, 1985, 1987). However, both total numbers of nevi of any type and total numbers of atypical nevi are associated with relative risks from 3 to 30-fold, with most results being in the area of 5 to 20-fold (Elwood *et al.*, 1986; Holman & Armstrong, 1984*b*; Roush *et al.*, 1985; Roush *et al.*, 1987; Roush *et al.*, 1988; Roush *et al.*, 1988; Nordlund *et al.*, 1985; Green *et al.*, 1986; Holly *et al.*, 1986; Rhodes *et al.*, 1980). Thus, the epidemiologic studies would suggest that nevi (either total nevi or numbers of atypical nevi) are better predictors of melanoma than freckling. If this is true, one possible explanation might be biologic (e.g., that nevi might be direct precursors of melanoma) (Roush, 1988; Roush *et al.*, 1988). However, the present results suggest another explanation, namely, that inconsistencies in categorisation of freckling may obscure an otherwise high relative risk for melanoma due to freckling.

While two previous studies have touched on the subject of inter-observer agreement on clinical diagnosis of nevi (MacKie *et al.*, 1985; Cooke, 1988) there are no concordance studies providing quantitative data on numbers of nevi. In a study of 432 healthy volunteers, MacKie and colleagues recorded the total number of nevi 3 mm or greater in diameter for each individual. Excellent inter-observer agreement between two dermatologists was reported (MacKie *et al.*, 1985) for nevus counts carried out in 20 subjects, but no quantitative data were provided. Cooke, Elder and Greene recorded rates of agreement on clinical diagnosis of nevi in a survey of melanocytic nevi in Milton, New Zealand (Cooke, 1988). The three observers reviewed 200 clinical photographs of flat, tan nevi 2 to 4 mm in diameter and concurred on a diagnosis of melanocytic nevus in 89% of the lesions. However, they did not study rates of agreement on total numbers of nevi.

Our results concerning counting of nevi indicate that all categories of nevi were reliably recorded by the examiners. In fact, except for one pair of examiners assessing palpable arm nevi, there was statistically-significant concordance among all pairs of observers for all counts of nevi (Table III). Similarly there was overall agreement among the correlations for the three examiners in Table IV. The highest correlation coefficients were recorded for counts of total body nevi (Tables III and IV). From these results we believe that in general nevi can be reliably counted.

Potential sources of misdiagnosis on clinical evaluation of nevi include simple lentigines (confusion with junctional or occasionally compound nevi), solar lentigines, and seborrheic keratoses. However, based on histologic evaluation of lesions removed, we have found that the latter categories of lesions account for about 6% of all pigmented lesions clinically diagnosed as melanocytic nevi (Barnill & Roush, 1990).

In conclusion, the present study has provided the first comprehensive evaluation of inter-clinician agreement on clinical assessment of freckling and quantification of nevi. Our findings have shown that numbers of nevi can be consistently recorded by physicians of varied medical background and experience. These results are also of considerable relevance to epidemiologic research concerning risk factors for cutaneous melanoma and also for comparison of results from different studies on this topic.

The contributions of Drs Paul Duray and Linda Titus-Ernstoff are greatly appreciated. We also thank Miss Michelle Wood for her efforts in the preparation of the manuscript.

### References

BARNHILL, R.L. & ROUSH, G.C. (1990). Histopathological Spectrum of clinically atypical melanocytic nevi: studies of non-familial melanoma II. *Arch. Dermatol.*, **126**, 1315.

BARNHILL, R.L., ROUSH, G.C. & DURAY, P.H. (1990). Correlation of histologic architectural and cytoplasmic features with nuclear atypia in atypical (dysplatic) nevomelanocytic nevi. *Hum. Pathol.*, **21**, 51.

BERAL, V., EVANS, S., SHAW, H. & MILTON, G. (1983). Cutaneous factors related to the risk of malignant melanoma. *Br. J. Dermatol.*, **109**, 165.

COOKE, K.R. (1988). Frequency of benign pigmented naevi in the general population. In *Elwood, Melanoma and Naevi: Incidence, Interrelationships and Implications*, pp. 8–26. Karger: Basel.

DUBIN, N., MOSENON, M. & PASTERNACK, B.S. (1986). Epidemiology of malignant melanoma: pigmentary traits, ultraviolet radiation, and the identification of high-risk populations. In *Epidemiology of Malignant Melanoma*, Gallagher, R.P. (ed.). pp. 56–75. Springer-Verlag: New York.

ELWOOD, J.M., WILLIAMSON, C. & STAPLETON, P.J. (1986). Malignant melanoma in relation to moles, pigmentation, and exposure to fluorescent and other lighting sources. *Br. J. Cancer*, **53**, 65.

GREEN, A., MACLENNAN, R. & SISKIND, V. (1985*a*). Common acquired naevi and the risk of malignant melanoma. *Int. J. Cancer*, **35**, 297.

GREEN, A., BAIN, C., MCLENNAN, R. & SISKIND, V. (1986). Risk factors for cutaneous melanoma in Queensland. *Recent Res. Canc. Res.*, **102**, 76.

HICKS, N., ZACK, M., CALDWELL, G.G. & MCKINLEY, T.W. (1985). Life-style factors among patients with melanoma. *South Med. J.*, **78**, 903.

HOLLY, E.A., KELLY, J.W., SHPALL, S.N. & CHIU, S.-H. (1986). Number of melanocytic nevi as a major risk factor for malignant melanoma. *J. Am. Acad. Dermatol.*, **15**, 705.

HOLMAN, C.D.J. & ARMSTRONG, B.K. (1984*b*). Pigmentary traits, ethnic origin, benign nevi, and family history as risk factors for cutaneous malignant melanoma. *JNCI*, **72**, 257.

KLEPP, O. & MAGNUS, K. (1979). Some environmental and bodily characteristics of melanoma patients. A case-control study. *Int. J. Cancer*, **23**, 482.

MACKIE, R.M., FREUDENBERGER, T. & AITCHISON, T.C. (1989). Personal risk-factor chart for cutaneous melanoma. *Lancet*, **ii**, 487.

MACKIE, R.M., ENGLISH, J., AITCHISON, T.C., FITZSIMONS, C.P. & WILSON, P. (1985). The number and distribution of benign pigmented moles (melanocytic nevi) in a healthy British population. *Br. J. Dermatol.*, **113**, 167.

NORDLUND, J.J., KIRKWOOD, J., FORGET, B.M., SCHEIBNER, Z.A., ALBERT, D.M., LERNER, E. & MILTON, G.W. (1985). Demographic study of clinically atypical (dysplastic) nevi in patients with malenoma and comparison subjects. *Canc. Res.*, **45**, 1855.

RHODES, A.R., SOBER, A.J., MIHM, M.C. Jr & FITZPATRICK, T.B. (1980). Possible risk factor for primary cutaneous malignant melanoma (abstract). *Clin. Res.*, **28**, 252A.

ROUSH, G.C. (1988). Chapter 6. Abnormal nevi, excess total nevi and melanoma: an epidemiologic perspective. In *Malignant Melanoma: Biopsy, Diagnosis and Therapy*, pp. 85–100, 191–195. Kluwer: Boston.

ROUSH, G.C., HOLFORD, T.R., SCHYMURA, M.J. & WHITE, C. (1987). *Cancer Risk and Incidence Trends*. Hemisphere: Washington, DC.

ROUSH, G.C., NORDLUND, J.J., FORGET, B., GRUBER, S.B. & KIRKWOOD, J.M. (1988). Independence of dysplastic nevi from total nevi in determining risk for nonfamilial melanoma. *Prev. Med.*, **17**, 273.

ROUSH, G.C., TITUS, L.J., ERNSTOFF, M.S., BARNHILL, R.L., KLAUS, S., LERNER, A., DURAY, P.H. & STENN, K. (1985). Pigmentary risk factors for biopsy proven dysplastic nevi in determining risk for melanoma (abstract C77). *Proc. Am. Soc. Clin. Oncol.*, **4**, 20.

SWERDLOW, A.J., ENGLISH, J., MACKIE, R.M., O'DOHERTY, C.J., HUNTER, J.A.A., CLARK, J. & HOLE, D.J. (1986). Benign melanocytic nevi as a risk factor for malignant malanoma. *Br. Med. J.*, **292**, 1555.

ZAR, J. (1984). *Biostatistical Analysis*, pp. 323–325. Prentice-Hall, Inc: Second Edition.